# Insights from Regulatory Data on Development Needs of Community Pharmacy Professionals

**DOI:** 10.3390/pharmacy8030111

**Published:** 2020-07-07

**Authors:** Katherine Morris, Anita Arzoomanian

**Affiliations:** 1 Information and Data Management, Ontario College of Pharmacists, Toronto, ON M5R 2R4, Canada; 2 Registrant Competence, Ontario College of Pharmacists, Toronto, ON M5R 2R4, Canada; aarzoomanian@ocpinfo.com

**Keywords:** continuing professional development, continuing education, pharmacy education, pharmacy workforce development, pharmacy practice change management, scope of practice, remediation, competency gaps, health regulatory body

## Abstract

The aim of this study was to use data available to a Canadian health professions regulator (Ontario College of Pharmacists) to identify areas of opportunity where practitioners (pharmacists and pharmacy technicians) could benefit from further development, in order to optimize practice and improve the quality of care. Four de-identified datasets were used to extract themes from areas of jurisprudence (1969 exam records), member practice assessments (2610 records), pharmacy assessments (2024 records) and conduct (640 case records). Outcome measures included performance in examinations and assessments and competency gaps identified in conduct investigations. Thematic analysis of outcomes was done in two stages. First, the four outcomes were derived independently for each dataset. Second, the top five issues were extracted for each dataset. It was hypothesized that common themes in competency gaps across all four datasets would emerge from this top five selection. We found three main common areas of opportunity where practitioners could benefit from further development: patient assessment and safety; documentation; and ethical, legal and professional responsibilities.

## 1. Introduction

Professional regulators have a mandate to serve and protect the public interest by ensuring that the members of the profession they regulate provide ethical, safe and quality professional services. Health profession regulators fulfill this mandate through activities which fall under the following programs: registration/licensing, quality assurance and complaints/conduct/discipline [1]. As integral components to these activities, regulators often administer tests to their registrants at entry to practice, conduct assessments through quality assurance or assess adherence to standards of practice. In addition, they address conduct concerns and complaints against registrants and take appropriate action in cases of professional misconduct. However, to date, there seems to be a lack of research on how combining these types of data could produce a cohesive picture of challenges and opportunities, especially at the system level. The current paper aims to address this research gap by finding common themes of development needs for community pharmacy professionals in Ontario.

The objective of this paper is to use data available to the Ontario College of Pharmacists (OCP) from exams, assessments and regulatory activities to identify areas of opportunity where most practitioners (pharmacists and pharmacy technicians) could benefit from further development, in order to optimize practice and improve the quality of care. These areas include implementing standards of practice, compliance with policies and regulations, and meeting general competencies for practice as defined by the National Association of Pharmacy Regulatory Authorities (NAPRA) [2].

A health profession regulator generates and accumulates various forms of data through the activities and functions it performs to support the public protection mandate. What if different activities such as competence assessments show patterns similar to site assessments or complaints and conduct issues? Experienced regulatory staff may anecdotally identify certain issues as recurring themes, as information is being routinely collected, however the data is not often analyzed or used to inform future practice improvement.

Some health profession regulators have ventured into data analysis to explore the potential for this data to help fulfill the regulatory mandate. A recent study used machine learning to examine topics of fitness-to-practice cases involving various UK health professionals including pharmacists [3]. The study identified some overlap or commonalities across professions, it also concluded that each profession has different priorities which professional associations and educational organizations should be aware of and work to address. The UK General Chiropractic Council undertook an independent review of its fitness-to-practice cases to understand the themes arising from allegations made about chiropractors [4]. Other studies [5,6] used conduct data to identify characteristics and risk factors affecting pharmacists subjected to disciplinary action.

Data analyzed need not be from only one area, such as fitness-to-practice or conduct. Regulators could combine data sources from other areas, such as quality assurance and routine audits to identify any common themes to inform and guide them in their regulatory mandate.

Continuing professional development is a critical component for pharmacy professionals to maintain competency to practice safely and provide quality patient care. Quality assurance programs administered by pharmacy regulators require members to maintain competence through continuous engagement in professional development, for example requiring pharmacists to conduct a self-assessment of their own learning needs [7]. These self-assessments typically focus on specific therapeutic areas, and it is not unusual for pharmacists to self-assess a need to pursue development and further training in emerging practice areas. New and emerging therapeutic drug categories are often identified as areas of opportunity to pursue continuing education. On the other hand, practitioners may have difficulty self-identifying their true competence gaps and may lack skills in self-appraisal and self-identification of learning needs [8]. 

Pharmacy practice in Canada is based on a foundation consisting of standards of practice, a code of ethics, and regulations that define society’s expectation of a self-regulated health care professional and the professional responsibilities of that pharmacy professional [9]. If this foundation is not strong, then the delivery of pharmacy services cannot be optimized, regardless of how many therapeutically focused continuing education programs pharmacists engage in. 

## 2. Materials and Methods 

The study was conducted in accordance with the Declaration of Helsinki, and the protocol was approved by the OCP ethics committee (2020-01-OKDA) and this paper reports on aggregated, de-identified data from January 2018 to December 2019.

Population and datasets: data for members registered as community pharmacists and pharmacy technicians as well as community pharmacies was included for the quantitative analysis. Four de-identified datasets were extracted from the OCP’s databases, detailed below. 

### 2.1. Jurisprudence (JP) Exam Data for Applicants (Pharmacists and Pharmacy Technicians)

The JP exam is an entry-to-practice requirement for registration as a pharmacist or pharmacy technician in Ontario. It represents one assessment of the “Ethical, Legal and Professional Responsibilities” competency from the Professional Competencies for Canadian Pharmacists and Pharmacy Technicians at entry-to-practice [10]. The exam assesses a candidate’s knowledge of provincial and federal legislation affecting pharmacy practice in Ontario [10]. It is based on an examination blueprint with seven key sections to be tested (conditions for sale; narcotics/controlled drugs; filing and labeling; billing and pricing; pharmacy operations; college structures/entry to practice/scope of practice; ethics/standards/responsibilities) [10], and the relative weight of each of the topic areas (i.e., number of multiple choice questions within each section). The topics listed in the examination blueprint represent subject matter that the sections address, however exam performance data is not available at the topic level. The exam, which is identical for pharmacist applicants and pharmacy technician applicants, is offered four times per year, therefore the data represents a total of eight exam sittings. The rationale for the exam being the same for all applicants is that jurisprudence is focused on regulatory expectations including legislation and ethics for which both pharmacists and pharmacy technicians are held accountable in Ontario. Data was analyzed at the section level for exams taken during 2018 and 2019.

### 2.2. Practice Assessment Data for Pharmacists and Pharmacy Technicians

At OCP, practice assessments are a critical component of quality assurance since they evaluate whether an individual is meeting the profession’s standards of practice [1,11]. Assessments occur at the member’s practice location where an OCP practice advisor uses defined criteria to evaluate the registrant’s practice [1,11]. 

Practice assessments of pharmacists support their role as medication experts and clinical decision-makers on four key domains (patient assessment, decision making, documentation, communication/education) [11] and thirteen indicators (Figure 2). The assessment tool also provides information about recommendations for improvement in each competence domain (Figure 3). Typically, most of the activities and practice of Ontario pharmacists involve prescription medications, and nonprescription (OTC) medication sales and recommendations generally represent a small segment of overall practice. Thus, when assessing pharmacists, the main focus is practice pertaining to prescription medications. However, when OTC recommendations and communication are observed, the same key performance indicators are considered and assessed.

**Figure A1:**
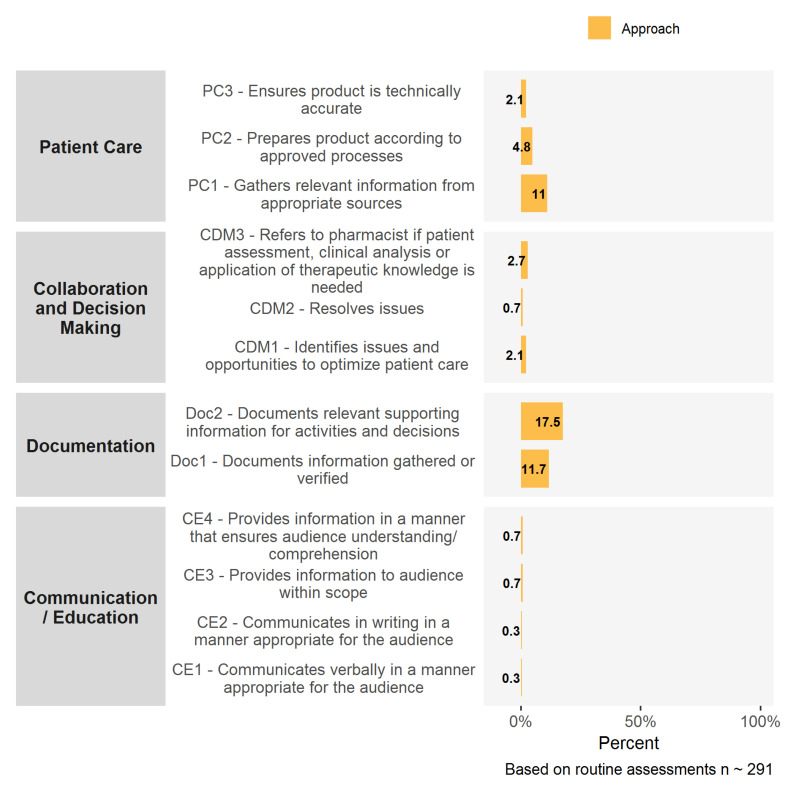
Gaps identified from assessments of pharmacy technicians during 2019.

**Figure A2:**
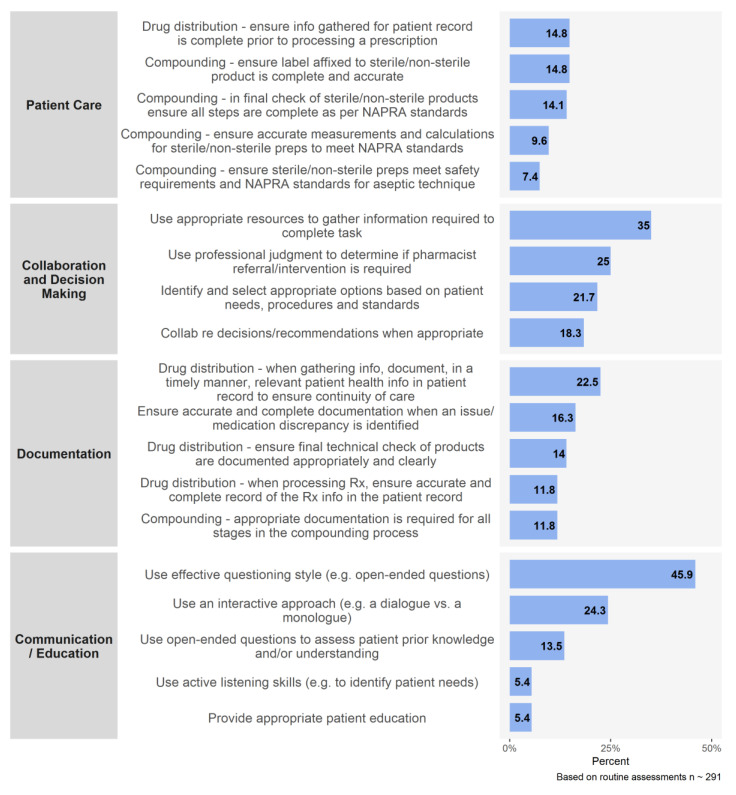
Top 5 recommendations from assessments of pharmacy technicians during 2019.

Practice assessments of pharmacy technicians support their technical role within the pharmacy on four domains (patient care support activities, collaboration and decision making, documentation, communication and education) [12] and twelve indicators (Figure A1), as well as recommendations for improvement in each domain (Figure A2). Technician assessments were rolled out in 2019 and were voluntary. 

The overarching intent of assessments is to evaluate the practitioners’ processes for each of the key performance indicators (Figure 2 and Figure A1) that can be rated as meeting standards, approaching standards or below standards. In order to identify competence gaps, we analyzed the data generated from assessments performed during 2018 and 2019 and focused on ratings where standards were not met. Different individuals were assessed each year. 

### 2.3. Operational Assessment Data for Community Pharmacies

Pharmacy assessments are conducted to ensure pharmacies are adhering to standards and have the proper processes and procedures in place for providing safe and quality care. During a community pharmacy assessment, an OCP operations advisor reviews and assesses the pharmacy’s operations and processes to determine whether or not the pharmacy is operating safely and according to professional standards and regulatory expectations. The advisor discusses the assessment with the pharmacy’s designated manager who is responsible for managing the pharmacy [13]. Routine operational assessments are conducted at all community pharmacies at least every four years, however many pharmacies are assessed more frequently based on certain risk factors. The risk factors include the type of specialty service provided by the pharmacy (e.g., sterile or specialty compounding, methadone dispensing) and prior history of unsatisfactory assessments.

The pharmacy assessment tool and criteria consist of various sets of standards against which the pharmacy is assessed [14]. The operations advisor uses this tool to determine if the pharmacy meets each of the standards, or if further action is required. If the pharmacy does not fully meet a standard, that particular standard is documented as not fully met. For each standard statement, there are subcomponents which provide further details of the standard or what is required to meet the standard. For each standard that is not fully met, the operations advisor documents the specific subcomponent which describes a gap in the pharmacy to be rectified or addressed. Data generated from assessments performed during 2018 and 2019 were analyzed by focusing on standards that were not fully met. 

### 2.4. Conduct Data for Pharmacists and Pharmacy Technicians

As a health profession regulator, one of the ways in which the OCP protects the public safety is by managing reported concerns. The Inquiries, Complaints and Reports Committee (ICRC) [15] oversees all complaints and investigations into a professional’s conduct and competence [15]. It is one of the statutory committees required through legislation which support the College Board. The committee consists of elected pharmacists, publicly appointed members from the Board and appointed non-board committee members [15]. The ICRC meets in small groups (panels) to review case submissions from complainants and pharmacy professionals. The panels also identify and document specific competencies that they deem as gaps or areas of opportunity for the practitioner. The list of competencies was developed by the OCP’s professional development and remediation (PDR) framework and is mostly based on the NAPRA Competencies for Entry to Practice for Canadian Pharmacists [2].

Using this data, we present the most common gaps in competency pertaining to conduct cases, as identified by members of the panel reviewing and adjudicating the conduct cases, which are classified into relevant professional development and remediation categories (e.g., ethical, legal and professional responsibilities; quality and safety) [2]. It is important to acknowledge that the data is dependent on the judgment call of the specific set of panel members reviewing each case. At the time of this article submission, calibration exercises and measures to maximize consistency have not yet been feasible. Within the PDR framework, competency gaps are recorded based on the following hierarchy: PDR classifications -> PDR key competencies -> PDR enabling competencies. In this paper, we use the term “competency gap” to refer specifically to issues arising from conduct cases which have their own associated “developmental needs”. We analyzed data from conduct cases reviewed during 2018 and 2019.

Outcomes: the quantitative analysis is based on four outcome measures computed from distinct datasets. The first outcome was member performance in jurisprudence exams, which was measured by the mean score in each exam section. The second outcome was member performance in practice assessments, measured by the overall percentage of registrants not meeting standards of practice. The third outcome was performance in operational assessments, which was measured by the overall percentage of community pharmacies not meeting operational standards. The fourth outcome was the frequency of competency gaps, which was measured by the overall number of conduct cases that were allocated to specific categories. 

**Figure 1:**
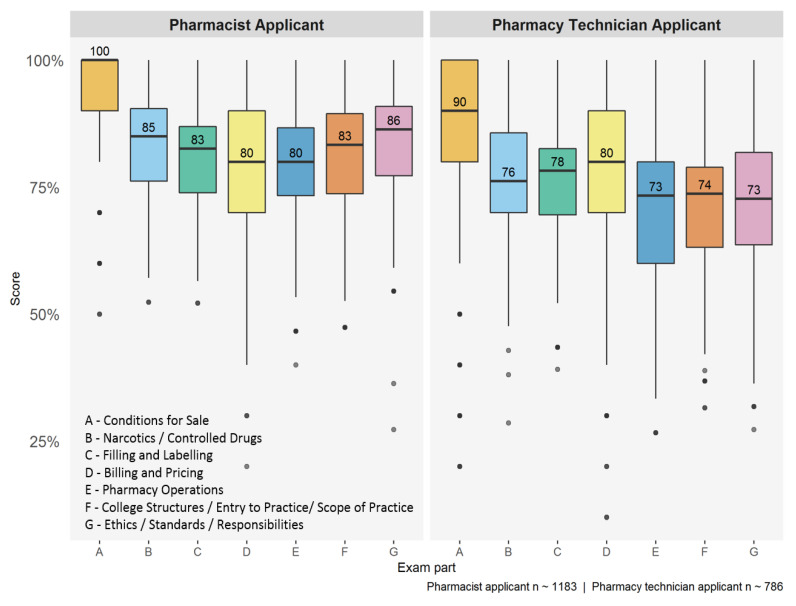
Distribution of jurisprudence (JP) exam scores by section during 2018 and 2019.

**Figure 2:**
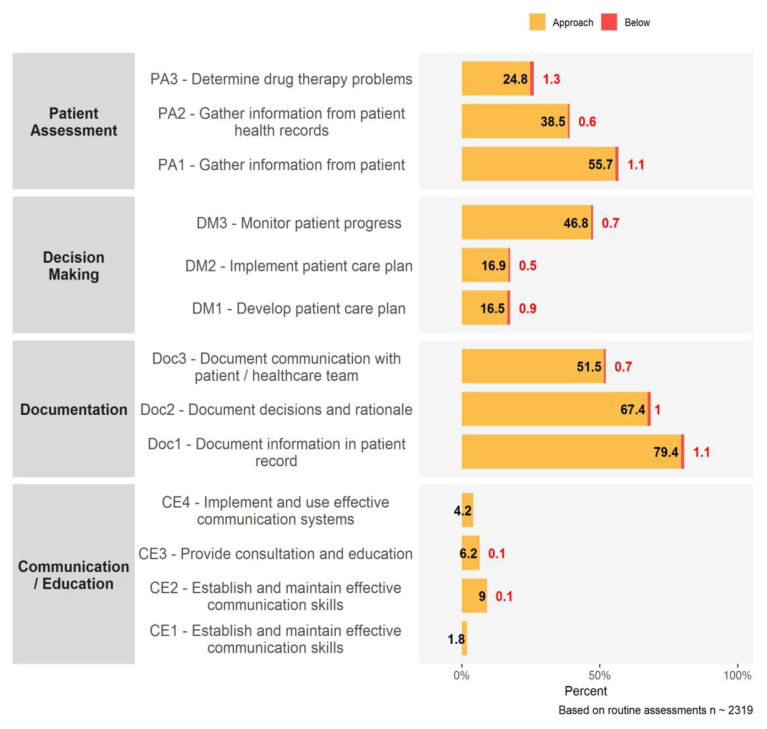
Gaps identified from assessments of community pharmacists during 2018 and 2019.

**Figure 3:**
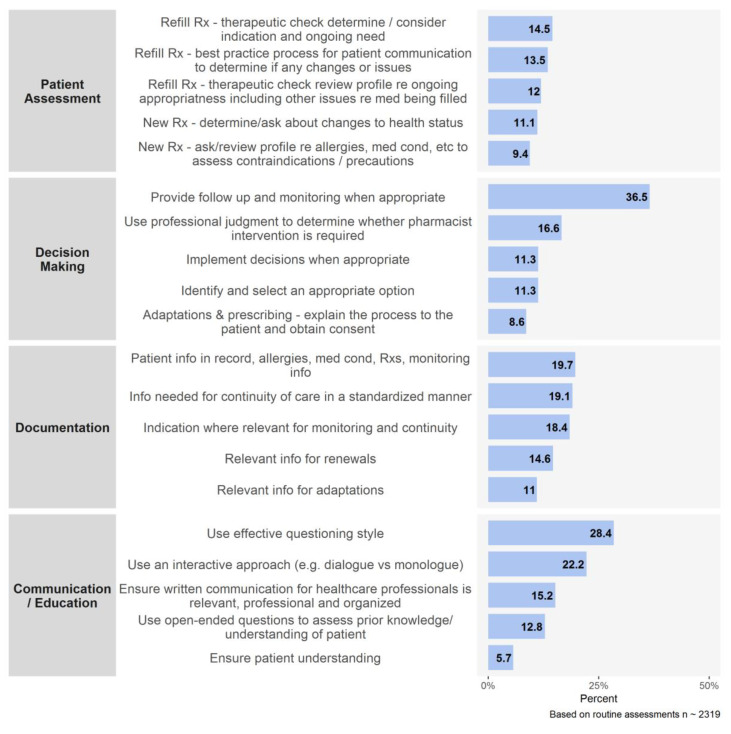
Top 5 recommendations from assessments of community pharmacists during 2018 and 2019.

**Figure 4:**
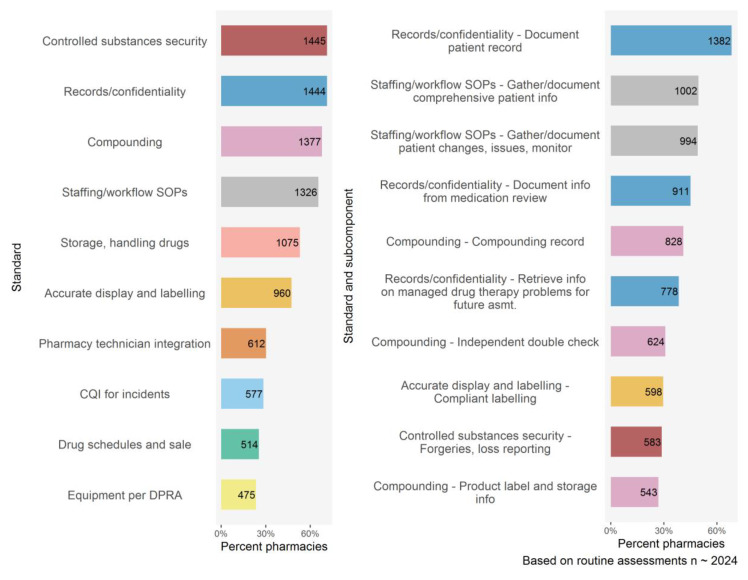
Top 10 gaps identified from operational assessments during 2018–2019.

**Figure 5:**
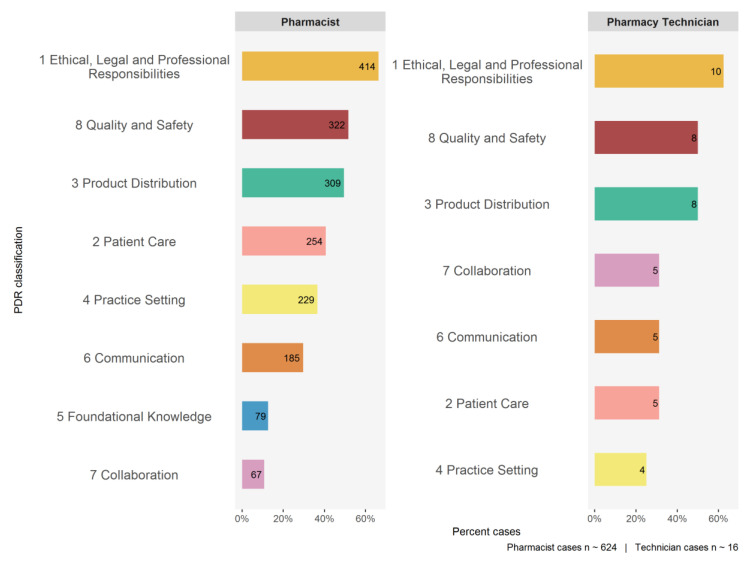
PDR classification gaps identified through ICRC cases during 2018 and 2019.

**Figure 6:**
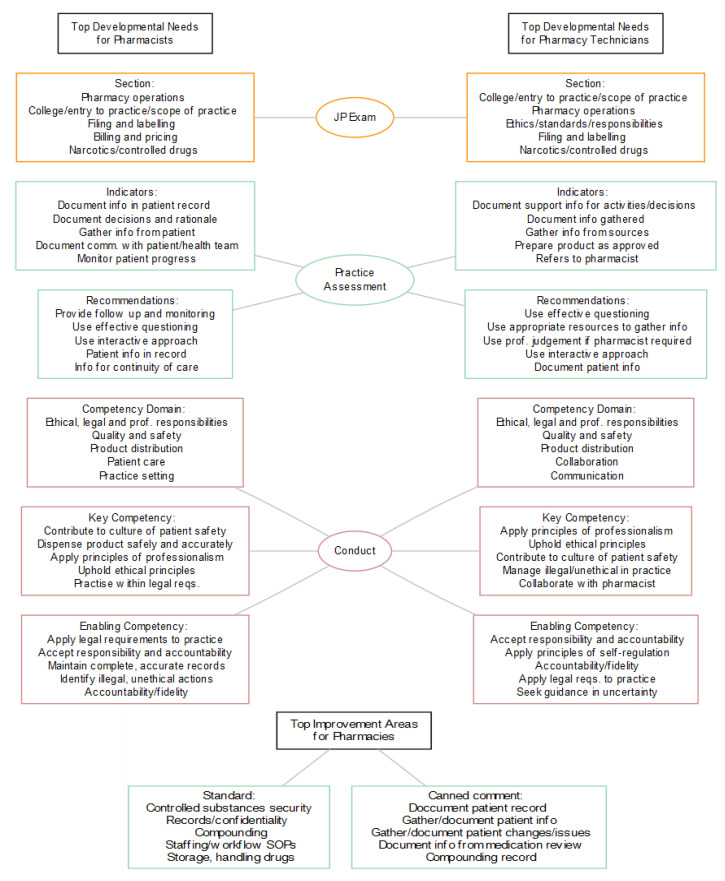
Top developmental needs identified from all data sources.

**Figure A3:**
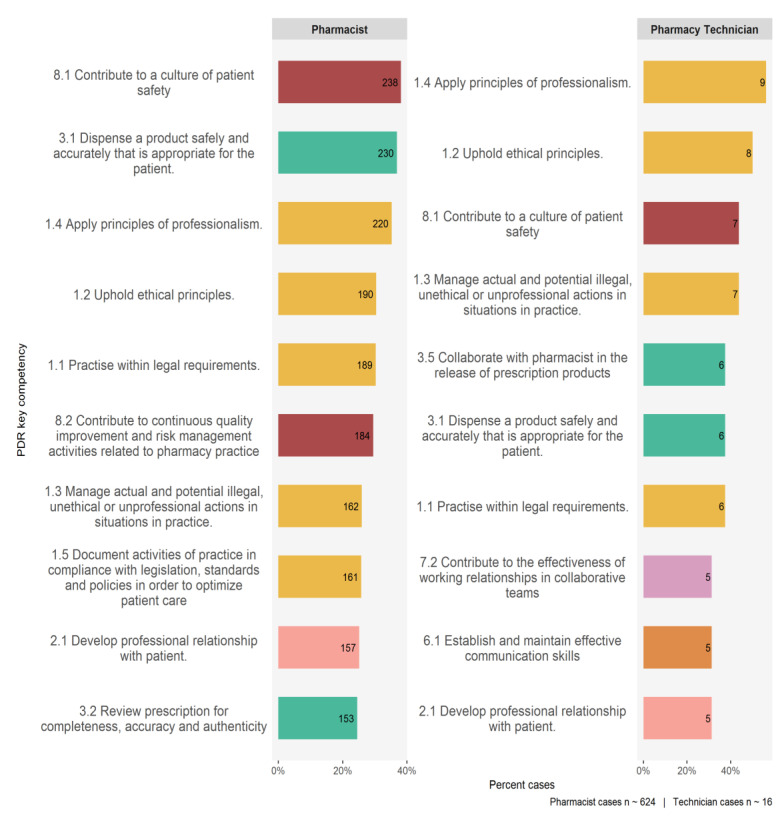
Top 10 PDR key competency gaps identified through Inquiries, Complaints and Reports Committee (ICRC) cases during 2018 and 2019.

**Figure A4:**
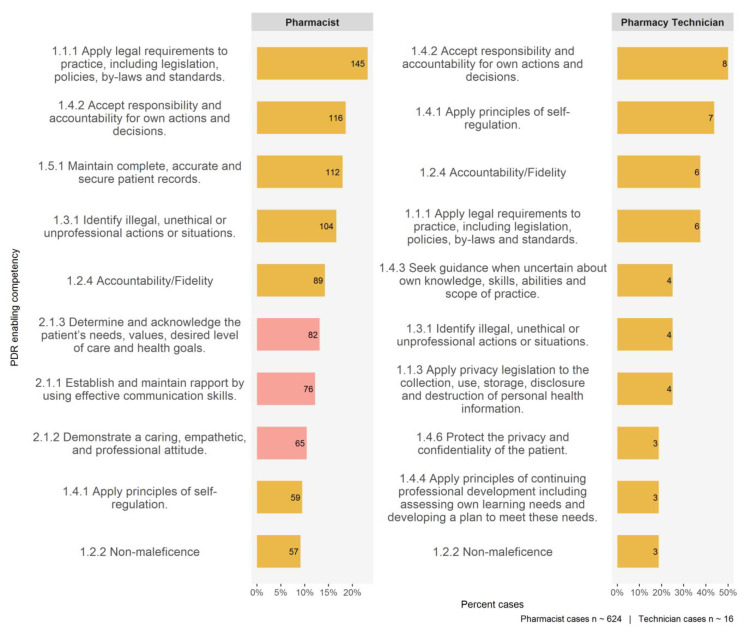
Top 10 PDR enabling competency gaps identified through ICRC cases during 2018 and 2019.

Analysis: all data analysis and visualization was done with the R software package (R Foundation for Statistical Computing, Version 3.6.2). Some datasets were only available for 2018 and 2019, thus for consistency we chose to focus all the analysis on this time period. Thematic analysis of outcomes was done in two stages. First, the four outcomes were derived independently for each dataset (Figures 1–5 and Figure A1, Figure A2, Figure A3 and Figure A4). Second, the top five issues were extracted for each dataset (Tables 1–5). It was hypothesized that common themes in developmental needs across all four datasets would emerge from this top five selection (Figure 6). Throughout the paper “developmental needs” is used as an umbrella term for issues arising from all four datasets (JP exams, practice and operational assessments and needs arising from conduct issues). We found three main common areas of opportunity where practitioners could benefit from further development: patient assessment and safety; documentation; and ethical, legal and professional responsibilities.

## 3. Results

The analysis in this section follows a chronological flow of observations for members, from entry to practice data (jurisprudence exams), followed by actual practice and operational assessments data, to conduct data. This method depicts a natural pathway of a pharmacy professional throughout their career.

### 3.1. Jurisprudence Exam

During 2018 and 2019, there were 1183 pharmacist applicants and 786 pharmacy technician applicants that took the JP exam. The boxplot with overall results by section, and their respective medians, is shown in Figure 1.

A more detailed summary of exam scores by member type and section is given in Table 1. We used bootstrapped confidence intervals for the mean scores based on the bias corrected, accelerated (BCa) method [16,17]. For pharmacist applicants, the sections with the lowest mean scores were related to pharmacy operations (79.5%) and scope of practice (81.4%) as well as filling and labeling (81.5%). For pharmacy technician applicants, the sections with the lowest mean scores arose from scope of practice (71.4%), pharmacy operations (71.6%) and ethics/standards/responsibilities (72.4%).

### 3.2. Practice Assessments

For pharmacists, there were 2319 assessments done during 2018 and 2019 and the gaps expressed as percentages for each indicator are presented in Figure 2. We analyzed this data and focused on the top five recommendations within each domain, as detailed in Figure 3. The indicators with the highest proportions of competence gaps and the most frequent recommendations were related to gathering and documenting information about a patient (Table 2).

For pharmacy technicians, we analyzed the data generated from 291 assessments performed during 2019. However, the technician assessments were voluntary and this could explain why their performance (Figure A1) is overall better when compared to that of pharmacists (Figure 2). The indicators with the highest proportions of competence gaps and the most frequent recommendations (Figure A2) were related to documenting information about a patient (Table 3). 

### 3.3. Operational Assessments

During 2018 and 2019, there were 2024 routine community pharmacy assessments undertaken. The data set combines assessments results from all pharmacies assessed regardless of whether the pharmacies provided specialty services or had prior unsatisfactory assessments. The top ten operating standards that were not fully met and the top ten issues arising from subcomponents appear in Figure 4. The most prevalent themes (Table 4) arising from the analysis center around gathering and maintaining accurate and comprehensive information about patients, as well as handling and storing controlled substances.

### 3.4. Conduct

During 2018 and 2019, the ICRC reviewed 624 cases involving pharmacists and 16 cases involving pharmacy technicians. Each case can have multiple classifications, key competencies and enabling competencies, thus the categories shown in Figure 5, Figure A3 and Figure A4 are not mutually exclusive and the percentages do not add up to 100. 

**Figure A5:**
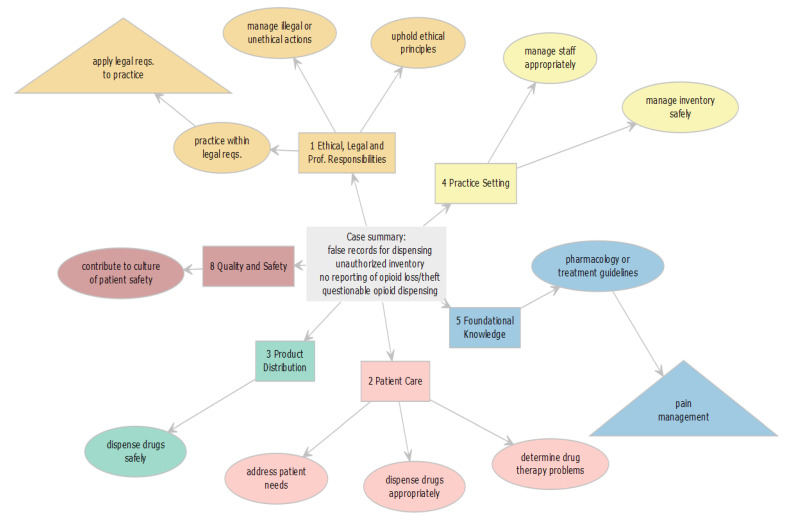
PDR gaps identified through a specific ICRC case (boxes denote PDR classifications; ovals denote PDR key competencies; triangles denote PDR enabling competencies).

First, we analyzed pharmacist cases, starting with their distribution among the first level of the PDR framework hierarchy (classifications, Figure 5), then we considered the second level (key competencies, Figure A3), and lastly we evaluated the third level (enabling competencies, Figure A4). For example, Figure A5 illustrates how one individual pharmacist’s case can link to multiple gaps in competence. The case summary was extracted from OCP’s public register [18]. The main themes emerging from analyzing the most frequent categories assigned to cases are shown in Table 5.

Second, we analyzed pharmacy technician cases using a similar method to pharmacists. Although there were far fewer technician cases reviewed by ICRC, we saw competency gap themes similar to those in pharmacist cases arising through the PDR framework (Figure 5, Figure A3 and Figure A4).

Finally, we can use data visualization to combine the top five developmental needs from each of the datasets (Figure 6). This enables easier comparison and extraction of commonalities. 

## 4. Discussion

In order to understand the main developmental needs emerging from all datasets, we extracted the top five gaps from each dataset analysis in the previous section (Figure 6). Thus, three common themes were revealed: patient assessment and safety; documentation; and ethical, legal and professional responsibilities.

Pharmacy practice has been evolving over the past half century to include an increased attention to patient-focused care, rather than mainly being a product-focused drug distribution practice. In the mid-1990s the pharmacy profession arrived at the consensus that pharmaceutical care rather than drug distribution alone should be the way of practice for pharmacists. As a result, faculties of pharmacy, given their responsibility for the education of students for practice, began developing curricula to prepare graduates capable of providing pharmaceutical care [19].

The Canadian Council for Accreditation of Pharmacy Programs (CCAPP) [20] has set professional qualifications and educational standards against which faculties of pharmacy are evaluated and accredited. With the evolution of practice towards pharmaceutical care and patient-centered care, the CCAPP standards have evolved to include educational outcomes that include a graduate being capable of performing the pharmacist patient-care process (information gathering, assessment, making a decision on a plan, implementing the care plan, follow up, collaboration, documentation, and communication) [20]. As a result, more recent pharmacy graduates have received instruction for these skills, whereas less recent graduates have not benefited from having these areas incorporated into their curricula. 

In 2009, NAPRA developed and released the Model Standards of Practice for Canadian Pharmacists [21] as standards of practice which all licensed pharmacists are expected to meet. These standards outline minimum expectations pertaining to how pharmacists are to apply their expertise in medications and medication use in their professional activities. These standards include those pertaining to patient assessment, decision making, documentation and communication as assessed in OCP’s practice assessments [11]. 

There has been limited research into the reasons why the areas of patient assessment, specifically gathering information for assessment, as well as documentation continue to be challenges in community pharmacy practice. Gaps in gathering information were not uniquely noted among pharmacists, but were also observed among pharmacy technicians. Documentation represented the largest gap area in practice assessments for both pharmacists and pharmacy technicians. Are these challenges partially due to a lack of engagement or commitment? Are they due to a lack of workflow and pharmacy software systems that are conducive to meeting these standards? Is lack of time the culprit? Or is the culprit a combination of the above? More research is needed to better understand pharmacy professionals’ behavior and engagement in their practice. Further work is needed even to understand how peak performance in community pharmacy practice is demonstrated and sustained [22]. One way of measuring optimal performance in pharmacy practice could be through quality indicators of pharmacy care at the system level. The first set of such quality indicators for community pharmacy were launched in 2019 by OCP, in partnership with Quality, Ontario Health (Q, OH) [23]. These indicators are intended to evaluate the quality of pharmacy practice based on the health outcomes of Ontario patients [23]. Other research has demonstrated that changing regulations alone does not always motivate pharmacists to adopt new practices [24]. The adoption of new practices in and of itself does not necessarily constitute high performance, unless it results in improved health outcomes for patients. In an effort to identify barriers to pharmacy professionals meeting standards of practice for patient assessment and documentation, all factors including individual competence, the work environment and workflow, as well as pharmacy management systems and technology need to be considered. 

Pharmacy education has evolved to adapt to the evolution in practice. Pharmacists who graduated prior to this evolution did not receive training and education on the current standards of practice pertaining to patient assessment or on appropriate documentation methods. Continued individual competence develops from initial skills training as well as regular application of those skills in practice. Therefore even pharmacists who received their education more recently may not have had the opportunity or encouragement in their work environment to put these skills into practice, if their mentors, preceptors, and supervisors did not provide the necessary role modelling. Pharmacists have heard many messages on why practice needs to change, however we often hear that they need to be shown how to change [24]. When training is not incorporated into routine practice, confidence and competence in these skills can suffer. The role and influence of preceptors, mentors and peers in modelling practice that meets standards must be considered for practitioner continuous professional development.

Information gathering for patient assessment requires interacting with the patient or their agent to gather relevant information. Although innovative community pharmacy workflow designs have emerged mostly in independently owned pharmacies, the current workflow design in many medium-to-high-volume community pharmacies places the pharmacist near the end of workflow at which point the prescription has already been processed and prepared. For the pharmacist to conduct a therapeutic check of appropriateness, the patient assessment often requires obtaining relevant information from the patient or their agent which is not well-facilitated or encouraged by current workflow setups and environments. 

There has been some effort in identifying barriers to meeting documentation standards, which include time (or lack thereof), lack of standardized templates, support through technology and software and confidence [25]. Pharmacy care is no longer meant to be based on a single drug dispensing interaction, but rather to include several interactions with a patient over time. On each occasion when a patient accesses care, current pharmacist standards of practice require an assessment of the patient and their prior health records. Hence, patient assessment is partly dependent on access to useful documentation. 

Traditional electronic practice management systems utilized in community pharmacies have developed to meet the business and regulatory requirements associated with the activity of dispensing, but are not as efficient in enabling or facilitating the documentation and exchange of information used by pharmacy professionals for patient-focused care. To address this issue, NAPRA approved and released a requirements document in 2013 [26]. Most, if not all, of these requirements have been supported by major software vendors of pharmacy practice management systems (PPMS) through features and system capabilities offered to users. However, two requirements that align with the standards of practice assessed in practice are yet to be implemented and utilized consistently and fully by pharmacies, namely:

- Requirement 10—Comprehensiveness of Clinical Records and Requirement [26] calls for all PPMS to provide users with the ability to create, access and update records of assessment, care plans, interventions and follow up by pharmacy professionals and all information indicated in provincial pharmacy standards of practice; and
- Requirement 17—Prescription Indications [26] necessitates all PPMS to provide users with the ability to input an indication or treatment objective for each prescription.

Even when a PPMS vendor has incorporated all NAPRA requirements, including requirements 10 and 17, these features may not be utilized by pharmacies if they are not recognized by practitioners and management as important tools for practice. Pharmacists and pharmacy managers who have managed without these tools in the past, may not realize the necessity and benefit of these features to quality patient care.

These requirements, if implemented and used consistently by pharmacies and pharmacists through consistent standard operating procedures, would directly facilitate the key performance indicators of the documentation domain in practice assessments which pharmacists did not fully meet (Table 2). Similarly, they would aid community pharmacies in meeting operational standards of record keeping/confidentiality and their related canned comments regarding documentation of patient record and information from medication reviews (Table 4).

In addition, the work environment, systems and technology, as well as workflow have not evolved enough to facilitate and enable patient care documentation and charting. As a result, graduates who received instruction in documentation in their pharmacy education have had limited opportunities to practice their documentation skills in a manner that builds competence and becomes a natural component of practice. Pharmacists who have been practicing longer have articulated the need for additional training in documentation skills [27].

The work environment and workflow are key components of pharmacy operations. We note that in the jurisprudence exam the section on pharmacy operations has one of the lowest mean scores for both pharmacist and pharmacy technician applicants. The two groups also share the section on scope of practice as a section with lowest mean scores. Clarity around scope of practice is essential for effective workflow and collaboration between pharmacists and pharmacy technicians.

Another theme revealed primarily in the conduct data is quality and patient safety. Many of the cases in this analysis are the result of medication incidents affecting patient safety. The contributing factors to these incidents can consist of themes identified in other data sources. There is often a link between whether the pharmacy or pharmacist met standards in patient assessment and whether a medication error occurred. Documentation according to standards can also play a key role in preventing medication incidents. Pharmacy operations that support effective documentation of pertinent information in the patient record for continuity of care could have avoided some of the quality and safety issues. In addition, organized staffing and workflow which enable pharmacists to meet the standards of practice can prevent certain medication incidents as part of a systems focused quality improvement process. 

Finally, another main theme of developmental needs which emerged ties in directly to NAPRA’s first-listed competency domain, namely ethical, legal and professional responsibilities [2], for both pharmacists and pharmacy technicians. In addition to the legal obligations and rules that must be followed, the competencies include ethical obligations and adherence to the profession’s code of ethics. They encompass professional responsibilities as a self-regulated health care professional, a concept which is often difficult to teach and define, yet fundamental to understanding the pharmacy professionals’ obligation to society. The competencies in this domain are mainly assessed at entry to practice in the jurisprudence exam and later identified if the pharmacy professional is a subject of a conduct concern. In general, the professionals who have had a conduct concern need a clearer and more solid grasp of their professional responsibilities and obligations. Continuing education programs which incorporate ethical and professional responsibilities can assist in reminding professionals of their obligations and clarifying society’s expectation of them as a self-regulated professionals.

There are strengths and limitations when interpreting the results of the analysis. The datasets were large enough to derive robust conclusions (e.g., 1969 JP exam records for applicants; 2610 practice assessment records for pharmacists and pharmacy technicians; 2024 operational assessment records for pharmacies; 640 conduct case records for pharmacists and pharmacy technicians). Practice and operational assessment data included two distinct groups of members and pharmacies which were assessed in each year. This added validity and reliability to the findings derived from the datasets.

One caveat, related to the conduct data, is that panel decisions are based on judgement calls (different panels may determine more or fewer gaps from the same case), so it is challenging to draw strong conclusions from cases and associated competency gaps. Efforts are underway to improve the consistency of ICRC gap assignment by introducing panel calibration training and adopting the NAPRA competency gaps as of 2020. Another caveat, related to the pharmacy technician data, is that their 2019 practice assessments were done on a voluntary basis. Thus, the performance results do not reflect a random sample, and could be exposed to self-selection bias.

## 5. Conclusions

Combining various regulatory datasets was a useful strategy to derive common themes of developmental needs for community pharmacy professionals. The work described in the article represents a novel approach to identifying continuing professional development needs of pharmacy professionals through the public protection lens, rather than relying solely on professionals’ self-appraisal of needs.

In terms of future work, it could be useful to create a member cohort with elements from all regulatory data sources available, including demographic information such as age, education and registration pathways, and practice characteristics. This could reveal new patterns and trends, and allow us to understand and address the developmental needs for population subgroups using a more tailored approach. One challenge with this approach would be having a large-enough sample in the member cohort, with sufficient common data points, to draw meaningful conclusions.

## Figures and Tables

**Table 1:** Jurisprudence exam scores summary (section names are taken from [10]).

Member Type	Exam Section	Median Score (%)	Mean Score (95% CI)
Pharmacist applicant	A: Conditions for Sale B: Narcotics/Controlled Drugs C: Filling and Labeling D: Billing and Pricing E: Pharmacy Operations F: College Structures/Entry to Practice/Scope of Practice G: Ethics/Standards/Responsibilities	100.0 85.0 82.6 80.0 80.0 83.3 86.4	93.4 (92.9, 93.9) 83.1 (82.6, 83.6) 81.5 (81.0, 82.0) 81.9 (81.1, 82.7) 79.5 (78.8, 80.2) 81.4 (80.8, 82.0) 83.3 (82.8, 83.9)
Pharmacy technician applicant	A: Conditions for Sale B: Narcotics/Controlled Drugs C: Filling and Labeling D: Billing and Pricing E: Pharmacy Operations F: College Structures/Entry to Practice/Scope of Practice G: Ethics/Standards/Responsibilities	90.0 76.2 78.3 80.0 73.3 73.7 72.7	83.2 (82.1, 84.3) 76.2 (75.5, 77.0) 75.9 (75.2, 76.6) 77.4 (76.3, 78.5) 71.6 (70.7, 72.5) 71.4 (70.6, 72.3) 72.4 (71.6, 73.2)

**Table 2:** Top gaps identified in pharmacist assessments done in 2018 and 2019 (domain, indicator and recommendation names are taken from [11]).

Domain	Indicator/Recommendation	Assessments (%)
Patient Assessment	PA1—gather info from patient ^1^	56.8
Decision Making	DM3—monitor patient progress ^1^	47.5
	Provide monitoring and follow up ^2^	36.5
Documentation	Doc1—info in patient record ^1^	80.5
	Doc2—decisions and rationale ^1^	68.4
	Doc3—communication with patient/healthcare team ^1^	52.2
	Patient info in record (e.g., allergies, medical conditions) ^2^	19.7
	Info needed for continuity of care ^2^	19.1
Communication/Education	Use effective questioning style ^2^	28.4
	Use an interactive approach ^2^	22.2

^1^ denotes an indicator rated as approaching or below standards; ^2^ denotes a recommendation.

**Table 3:** Top gaps identified in pharmacy technician assessments done in 2018 and 2019 (domain, indicator and recommendation names are taken from [12]).

Domain	Indicator/Recommendation	Assessments (%)
Patient Care	PC1—gather info from appropriate sources ^1^	11.0
	PC2—prepare product according to approved processes ^1^	4.8
Collaboration and Decision Making	CDM3—refer to pharmacist when needed ^1^	2.7
	Use appropriate resources to gather info required for task ^2^	35.0
	Use prof. judgement to determine if pharmacist intervention is required ^2^	25.0
Documentation	Doc1—document info gathered or verified ^1^	11.7
	Doc2—document supporting info for activities and decisions ^1^	17.5
	Document patient health info for continuity of care ^2^	22.5
Communication/Education	Use effective questioning style ^2^	45.9
	Use an interactive approach ^2^	24.3

^1^ denotes an indicator rated as approaching or below standards; ^2^ denotes a recommendation.

**Table 4:** Operational assessments summary of unmet criteria (standard and subcomponent descriptions are taken from [14]).

Standard and Subcomponent	Unmet (%)
Compounding	68.0
Full compounding record must be present	40.9
Controlled substances security	71.4
Records/confidentiality	71.3
Document patient record	68.2
Document information from medication review	45.0
Staffing/workflow SOPs	65.5
Gather and document comprehensive patient info	49.5
Gather and document changes to patient info	49.1
Storage, handling drugs	53.1

**Table 5:** Pharmacist competency gaps identified through ICRC cases (descriptions are taken from [2]). PDR: professional development and remediation.

Category	Description	Cases (%)
PDR classification	Ethical, Legal and Professional Responsibilities	66.3
	Quality and Safety	51.6
	Product Distribution	49.5
	Patient Care	40.7
	Practice Setting	36.7
PDR key competency	Contribute to a culture of patient safety	38.1
	Dispense a product safely and accurately	36.8
	Apply principles of professionalism	35.2
	Uphold ethical principles	30.4
	Practice within legal requirements	30.3
PDR enabling competency	Apply legal requirements to practice	23.2
	Accept responsibility and accountability for own actions and decisions	18.6
	Maintain complete, accurate and secure patient records	17.9
	Identify illegal or unethical actions or situations	16.7
	Accountability/fidelity	14.3

## References

[1] Winkelbauer S. (2020). An authentic, practice-based assessment as a catalyst for continuous professional development. Pharmacy.

[2] (2014). National Association of Pharmacy Regulatory Authorities (NAPRA). Professional Competencies for Canadian Pharmacists at Entry to Practice 2014.

[3] Hanna A., Hanna L. (2019). Topic analysis of UK fitness to practice cases: What lessons can be learnt?. Pharmacy.

[4] Williams S. (2014). Independent Review of General Chiropractic Council Fitness to Practise Cases 2010–2013.

[5] Phipps D.L., Noyce P.R., Walshe K., Parker D., Ashcroft D.M. (2011). Pharmacists subjected to disciplinary action: Characteristics and risk factors. Int. J. Pharm. Pract..

[6] Phipps D.L., Walshe K., Parker D., Noyce P.R., Ashcroft D.M. (2016). Job characteristics, well-being and risky behaviour amongst pharmacists. Psychol. Health Med..

[7] Ontario College of Pharmacists Self-Assessment Tool. https://www.ocpinfo.com/practice-education/qa-program/self-assessment/.

[8] Austin Z., Marini A., Macleod Glover N., Croteau D. (2005). Continuous professional development: A qualitative study of pharmacists’atitudes, behaviors, and preferences in Ontario, Canada. Am. J. Pharm. Educ..

[9] Ontario College of Pharmacists Self-Assessment Tool. https://www.ocpinfo.com/regulations-standards/professional-responsibility-in-practice/.

[10] Ontario College of Pharmacists Examination Blueprint. https://www.ocpinfo.com/registration/registration-requirements/jp-exam/jp-exam-blueprint/.

[11] Ontario College of Pharmacists Pharmacist Practice Assessment Criteria. https://www.ocpinfo.com/library/practice-related/download/PracticeAssessmentCriteria.pdf.

[12] Ontario College of Pharmacists Pharmacy Technician Practice Assessment Criteria. https://www.ocpinfo.com//library/practice-related/download/PharmacyTechnicianPracticeAssessmentCriteria.pdf.

[13] Ontario College of Pharmacists Designated Managers. https://www.ocpinfo.com/practice_resource/designated-managers/.

[14] Ontario College of Pharmacists Community Pharmacy Assessment Criteria. https://www.ocpinfo.com/wp-content/uploads/documents/CommunityPharmacyAssessmentCriteria.pdf.

[15] Ontario College of Pharmacists Committees. https://www.ocpinfo.com/about/council-committees/committees/.

[16] Carpenter J., Bithel J. (2000). Bootstrap confidence intervals: When, which, what? A practical guide for medical statisticians. Stat. Med..

[17] Efron B. (1987). Better bootstrap confidence intervals. J. Am. Stat. Assoc..

[18] Ontario College of Pharmacists Public Register. http://members.ocpinfo.com/tcpr/public/pr/en/.

[19] Perrier D.G., Winslade N., Pugsley J., Lavack L., Strand L.M. (1995). Designing a pharmaceutical care curriculum. Am. J. Pharm. Educ..

[20] The Canadian Council for Accreditation of Pharmacy Programs Accreditation-Standards for Canadian-First Professional Degree in Pharmacy Programs. http://ccapp-accredit.ca/wp-content/uploads/2016/01/Accreditation-Standards-for-Canadian-First-Professional-Degree-in-Pharmacy-Programs.pdf.

[21] National Association of Pharmacy Regulatory Authorities Model Standards of Practice for Canadian Pharmacists. https://napra.ca/sites/default/files/2017-09/Model_Standards_of_Prac_for_Cdn_Pharm_March09_layout2017_Final.pdf.

[22] Zubin A., Gregory P. (2020). Understanding psychological engagement and flow in community pharmacy practice. Res. Soc. Admin. Pharm..

[23] Ontario College of Pharmacists Quality Indicators for Pharmacy. https://www.ocpinfo.com/wp-content/uploads/2019/08/QualityIndicatorsLeaflet.pdf.

[24] Zubin A., Gregory P. (2019). Learning needs of pharmacists for an evolving scope of practice. Pharmacy.

[25] Canadian Pharmacists Association Documenting Pharmacy Interventions in a Busy Dispensary. https://www.pharmacists.ca/cpha-ca/assets/File/education-practice-resources/WebinarSlides-DocumentingInterventions.pdf.

[26] National Association of Pharmacy Regulatory Authorities Pharmacy Practice Management Systems: Requirements to Support NAPRA’s “Model Standards of Practice for Canadian Pharmacists”. https://napra.ca/sites/default/files/documents/NAPRA_Pharmacy_Practice_Management_Systems_November2013_b.pdf.

[27] Schindel T.J., Yuksel N., Breault R., Daniels J., Varnhagen S., Hughes C.A. (2019). Pharmacists’ learning needs in the era of expanding scopes of practice: Evolving practices and changing needs. Res. Soc. Admin. Pharm..

